# Supplementary material for: Reduced inter-subject functional connectivity during movies in autism: replicability across cross-national fMRI datasets
Source: Mol Autism. 2026 Feb 18;17:11. doi: 10.1186/s13229-026-00707-2 (PMC12922298; doi:10.1186/s13229-026-00707-2)
Supplement: Supplementary file 3 — Supplementary Material 3 [file 13229_2026_707_MOESM3_ESM.docx]

**Description of clinical scores:**

In the German data, all participants completed the Autism-spectrum Quotient (AQ) questionnaire (1), the Anticipatory and Consummatory Interpersonal Pleasure Scale (ACIPS) (2,3), the Liebowitz Social Anxiety Scale (LSAS) (4), the Toronto Alexithymia Scale (TAS-20) (5), the Beck’s Depression Inventory (BDI-II, German version) (6), the Reading the Mind in the Eyes test (RME) (7). Most German participants were assessed using the Autism Diagnostic Observation Schedule-2 (ADOS-2) – Module 4 (8) by experienced clinical psychologists. In the Finnish data, all participants completed the AQ questionnaire, the BDI-II (9), the Depression Anxiety Stress Scale-21 (DASS-21) (10), and the State-Trait Anxiety Inventory-X2 (STAI-X2) (11). ADOS-2 assessment was also performed by a trained clinical psychologist to quantify the autism severity in the autism group (12). All group scores described above are listed in Tables S1-4.

The ADOS assessment used in the German and Finnish datasets was based on the same version designed for adult autistic individuals, but administered in different languages. ADOS scores in both autism groups were significantly different from the within-site neurotypical group. However, a Mann-Whitney U test indicated that ADOS scores in the German autism group were significantly lower than those in the Finnish autism group (U = 63.5, *p* = 0.0018, *r* = -0.519). This difference may reflect variations in diagnostic criteria and procedures across countries. Conversely, AQ scores were significantly higher in the German autism group compared to the Finnish autism group (U = 342.0, *p* = 0.0001, *r* = 0.619). Since the AQ is a self-report measure, these results may be influenced by subjective bias. Cultural differences in self-perception may also play a role. However, these potential cross-cultural differences cannot be directly quantified within the current datasets.

**Table S1.** Clinical scores of the German Autism group.

|  | **ADOS** | **AQ** | **ACIPS** | **LSAS** | **TAS-20** | **BDI_II** | **RME** |
| --- | --- | --- | --- | --- | --- | --- | --- |
| **N** | 18 | 22 | 22 | 22 | 22 | 22 | 22 |
| **Mean** | 7 | 37.59 | 52.09 | 74.25 | 59.93 | 12.45 | 19.68 |
| **Std** | 3.83 | 7.96 | 13.20 | 21.57 | 7.88 | 9.71 | 4.81 |

**Table S2.** Clinical scores of the German Neurotypical group.

|  | **ADOS** | **AQ** | **ACIPS** | **LSAS** | **TAS-20** | **BDI_II** | **RME** |
| --- | --- | --- | --- | --- | --- | --- | --- |
| **N** | 14 | 25 | 25 | 25 | 25 | 25 | 25 |
| **Mean** | 0.78 | 13.68 | 84 | 20.96 | 40.68 | 4.12 | 25.44 |
| **Std** | 1.05 | 5.61 | 8.50 | 14.27 | 9.98 | 5.14 | 3.27 |

**Table S3.** Clinical scores of the Finnish Autism group.

|  | **ADOS** | **AQ** | **BDI-II** | **DASS-21** | **STAI-X2** |
| --- | --- | --- | --- | --- | --- |
| **N** | 18 | 18 | 18 | 18 | 18 |
| **Mean** | 12 | 27.44 | 8.22 | 14.88 | 43.83 |
| **Std** | 4.32 | 5.86 | 7.36 | 8.93 | 8.11 |

**Table S4.** Clinical scores of the Finnish Neurotypical group.

|  | **AQ** | **BDI-II** | **DASS-21** | **STAI-X2** |
| --- | --- | --- | --- | --- |
| **N** | 19 | 19 | 19 | 19 |
| **Mean** | 10.94 | 3.05 | 7.52 | 36.47 |
| **Std** | 3.53 | 2.91 | 5.96 | 6.33 |

**References**

1. Bishop DVM, Maybery M, Maley A, Wong D, Hill W, Hallmayer J (2004): Using self-report to identify the broad phenotype in parents of children with autistic spectrum disorders: A study using the Autism-Spectrum Quotient. *J Child Psychol Psychiatry* 45(8): 1431–1436. <https://doi.org/10.1111/j.1469-7610.2004.00325.x>
2. Gooding DC, Pflum MJ (2014a): The assessment of interpersonal pleasure: introduction of the anticipatory and consummatory interpersonal pleasure scale (ACIPS) and preliminary findings. *Psychiatry Res* 215: 237–243. <https://doi.org/10.1016/J.PSYCHRES.2013.10.012>
3. Gooding DC, Pflum MJ (2014b): Further validation of the ACIPS as a measure of social hedonic response. *Psychiatry Res* 215: 771–777. <https://doi.org/10.1016/j.psychres.2013.11.009>
4. Liebowitz MR (1987): Social phobia. Anxiety. *Mod Trends Pharmacopsychiatry* 22: 141-173. <https://doi.org/10.1159/000414022>
5. Bagby RM, Parker JDA, Taylor GJ (1994): The twenty-item Toronto Alexithymia Scale—I. Item selection and cross-validation of factor structure. *J Psychosom Res* 38(1): 23-32. <https://doi.org/10.1016/0022-3999(94)90005-1>
6. Besier T, Goldbeck L, Keller F (2008): Psychometrische Gütekriterien des Beck-Depressions-Inventars II (BDI-II) bei jugendpsychiatrischen Patienten [Psychometric properties of the Beck depression inventory-II (BDI-II) among adolescent psychiatric patients]. *Psychother Psychosom Med Psychol* 58(2): 63-68. <https://doi.org/10.1055/s-2007-986195>
7. Baron-Cohen S, Wheelwright S, Hill J, Raste Y, Plumb I (2001): The "Reading the Mind in the Eyes" Test revised version: A study with normal adults, and adults with Asperger syndrome or high-functioning autism. *J Child Psychol Psychiatry* 42(2): 241-251. <https://doi.org/10.1111/1469-7610.00715>
8. Hus V, Lord C (2014): The Autism Diagnostic Observation Schedule, Module 4: Revised Algorithm and Standardized Severity Scores. *J Autism Dev Disord* 44: 1996–2012. <https://doi.org/10.1007/s10803-014-2080-3>
9. Hautzinger M, Keller F, Kühner C (2009): *BDI II Beck Depressions-Inventar Revision*, *2nd ed.* Frankfurt am Main: Pearson Assessment & Information GmbH.
10. Lovibond SH, Lovibond PF (1995): The Structure of Negative Emotional States: Comparison of the Depression Anxiety Stress Scales (DASS) with the Beck Depression and Anxiety Inventories. *Behav Res Ther* 33(3): 335-343. <https://doi.org/10.1016/0005-7967(94)00075-U>.
11. Spielberger CD (1983): State-Trait Anxiety Inventory (Form Y). In: Spielberger CD, editior. *Manual for the State-Trait Anxiety Inventory*. Consulting Psychologists Press, pp 1-24.
12. Lord C, Rutter M, DiLavore PC, Risi S, Gotham K, Bishop S (2012): *Autism Diagnostic Observation Schedule,* 2nd ed. Log Angeles, CA: Western Psychological Corporation.

| **Clip_ID** | **Duration (s)** | **Category** | **Clip_ID** | **Duration (s)** | **Category** |
| --- | --- | --- | --- | --- | --- |
| 1 | 11 | non-interaction | 30 | 9,2 | neutral |
| 2 | 10,6 | social | 31 | 13,7 | non-interaction |
| 3 | 16,2 | emotion | 32 | 11 | non-interaction |
| 4 | 10,6 | non-interaction | 33 | 11,8 | non-interaction |
| 5 | 12 | social | 34 | 11 | non-interaction |
| 6 | 13,5 | neutral | 35 | 18,5 | social |
| 7 | 15 | pain | 36 | 10,6 | non-interaction |
| 8 | 16,1 | emotion | 37 | 10,7 | pain |
| 9 | 11,8 | emotion | 38 | 10,6 | neutral |
| 10* | 11,6 | pain | 39 | 12 | emotion |
| 11 | 9,9 | non-interaction | 40 | 14,6 | emotion |
| 12 | 13,6 | pain | 41 | 11,1 | social |
| 13 | 18 | neutral | 42 | 13,7 | non-interaction |
| 14 | 10 | emotion | 43 | 14,3 | social |
| 15 | 11,8 | social | 44 | 13,8 | emotion |
| 16 | 10,3 | neutral | 45 | 11,8 | non-interaction |
| 17 | 12,7 | pain | 46 | 18 | pain |
| 18 | 11 | emotion | 47 | 10,1 | pain |
| 19 | 10,2 | emotion | 48 | 10,7 | social |
| 20 | 15,6 | emotion | 49 | 16 | pain |
| 21 | 14,8 | social | 50 | 10,9 | non-interaction |
| 22 | 10,7 | non-interaction | 51 | 11,7 | non-interaction |
| 23 | 11,1 | social | 52 | 9,1 | pain |
| 24 | 11,2 | neutral | 53 | 11,9 | neutral |
| 25 | 10,4 | social | 54 | 16 | emotion |
| 26* | 11 | pain | *Two clips were deleted in the German dataset as required by clinicians. | | |
| 27 | 12,4 | neutral |  |  |  |
| 28 | 22,3 | emotion |  |  |  |
| 29 | 11,8 | neutral |  |  |  |

**Table S5.** Duration and categories of movie clips.

**Fig. S1** Count of subcortical regions with ISFC group difference (two directions, p < .01). Amyg: Amygdala; Hipp: Hippocampus; Str: Striatum; Tha: Thalamus; CER: Cerebellum. Details with region names and values are listed in Supplementary Table 7.

**
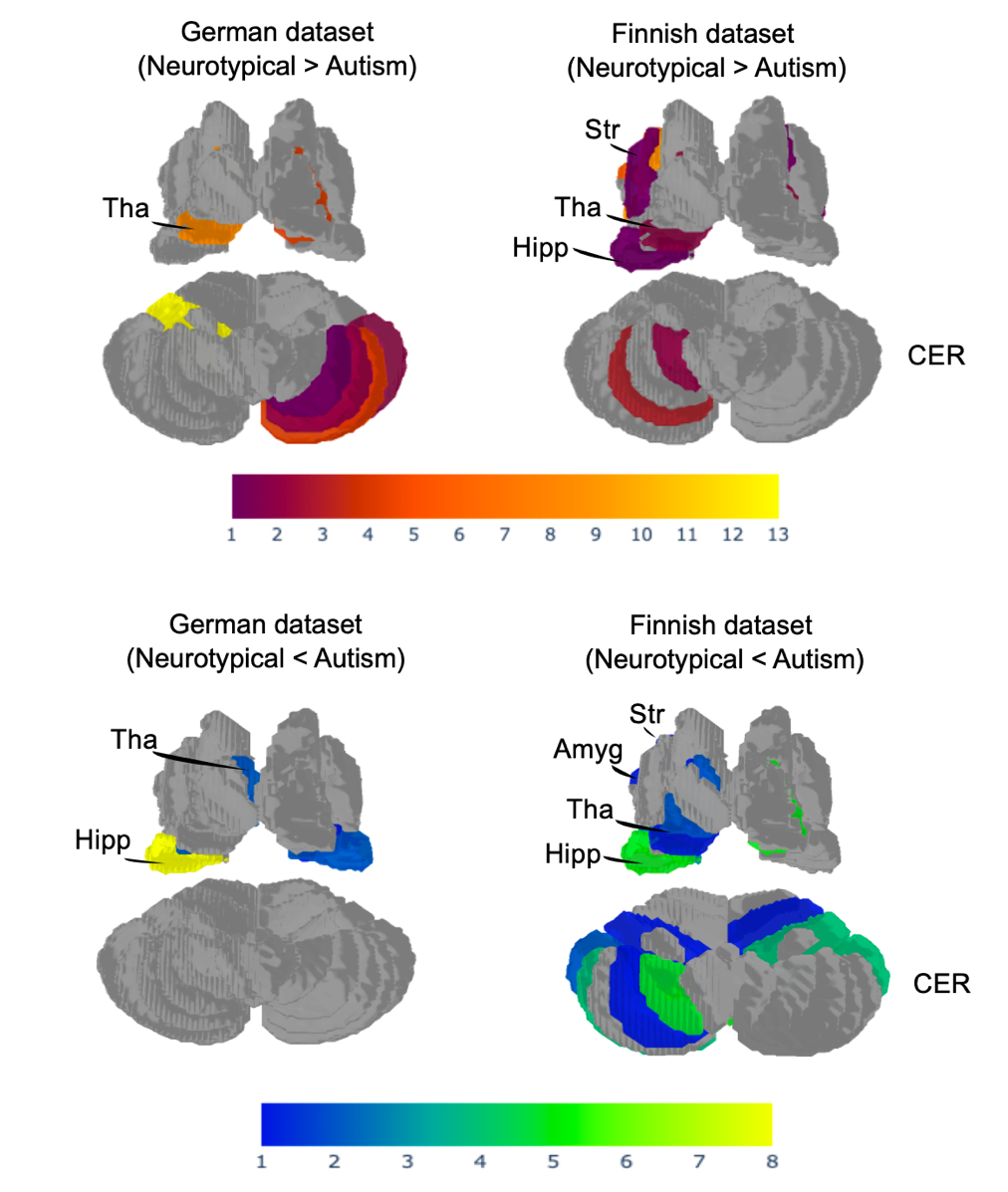
**

**ISFC group differences across sexes in two datasets**

We observed higher correlation and replication rates of pairwise ISFC group differences across sexes than across countries (Fig. S2). The replication rate between German males and females was 19% at an ISFC significance level of p < .05. ISFC differences showed significant correlations between German males and females (Pearson r = 0.201, p_permuted_ < .001) and between German males and Finnish males (Pearson r = 0.146, p_permuted_ < .001). However, German females showed a weak correlation with Finnish males (Pearson r = 0.022, p_permuted_ = .130). Overall, males exhibited higher ISFCs than females in both diagnostic groups. Notably, ISFC differences between neurotypical and autistic individuals in Finnish males more closely resembled those of German males than those of German females.

**
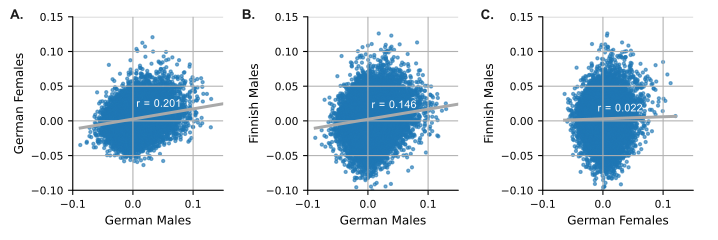
**

**Fig. S2** Scatterplots of pairwise ISFC difference (neurotypical-autism) across sexes in German and Finnish participants. (A) German males vs. German females. (B) German males vs. Finnish males. (C) German females vs. Finnish males. The ROI orders were randomly permuted 5000 times to generate the null distribution for the Pearson correlations between different groups.

**Effects of age and sex on the ISFC group differences**

The age distribution in the German and Finnish datasets shows slight differences, with the German dataset having a higher mean and standard deviation. Independent t-tests revealed no significant age differences between neurotypical and autism groups within each dataset, nor between the neurotypical groups across datasets. However, the German autism group was significantly older than the Finnish autism group (t = 3.360, *p* = 0.0019).

To evaluate potential confounding effects of age and sex on ISFC group difference, linear regression was used to remove these effects (where available) from the ISFC matrices. In the Finnish dataset, available confounders included pairwise mean age and absolute age difference. In the German dataset, sex was included as an additional confounder. All confound variables were z-scored, and a constant term was added. For each ROI pair, linear models were fit across subject pairs, and confound-related variance was regressed out, resulting in cleaned ISFC matrices that are orthogonal to age and sex.

Next, group-level median ISFC matrices were computed for neurotypical and autism groups, both before and after confound regression. No notable changes were observed. This was confirmed by a Mantel test comparing pre- and post-regression difference matrices, which showed a strong correlation (r = 0.98, *p* < .001, 5000 permutations), indicating that diagnostic effects were not primarily driven by age- or sex-related confounds (Fig. S3).


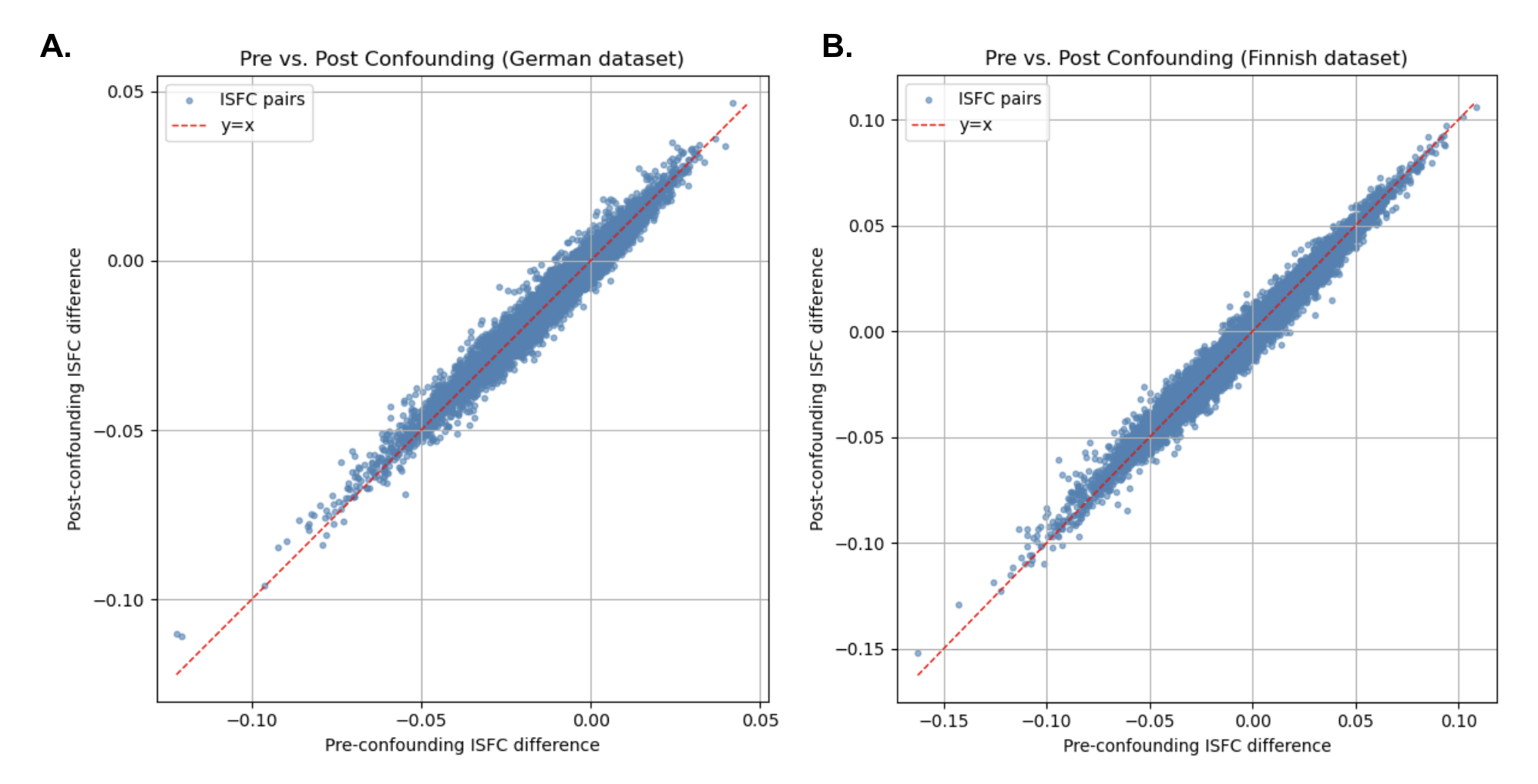


**Fig. S3** Scatterplots comparing group differences in ISFC before and after confound regression for the (A) German and (B) Finnish datasets. Each datapoint represents a median pairwise ISFC group difference (Neurotypical - Autism). The x-axis shows the uncorrected group difference, while the y-axis reflects the group difference after regressing out relevant confounds. A diagonal line is plotted in red for comparison of pre- and post-regression values.

**Effects of education levels on the ISFC group differences**

Previous studies have indicated that socioeconomic status (SES) influences cognitive and socio-emotional development (1,2). Therefore, we additionally evaluated the effects of the available related data (i.e. education levels in the Finnish dataset) on ISFC group differences.

In the Finnish dataset, we have three levels of education across subjects: (1) primary school; (2) secondary school, high school, vocational school, (3) university, university for applied science. We used linear regression to remove the effects of education levels from the ISFC matrices. Pairwise sums and differences of the education level across subjects were included as confounders. All confound variables were z-scored, and a constant term was added. For each ROI pair, linear models were fit across subject pairs, and confound-related variance was regressed out, resulting in cleaned ISFC matrices that are orthogonal to education levels. Group-level median ISFC matrices were computed for neurotypical and autism groups, both before and after confound regression. No notable changes were observed. This was confirmed by a Mantel test comparing pre- and post-regression group difference matrices, which showed a strong correlation (r = 0.924, p < .001, 5000 permutations), indicating that group effects were not primarily influenced by individual differences in education levels in the Finnish dataset (Fig. S4).

*
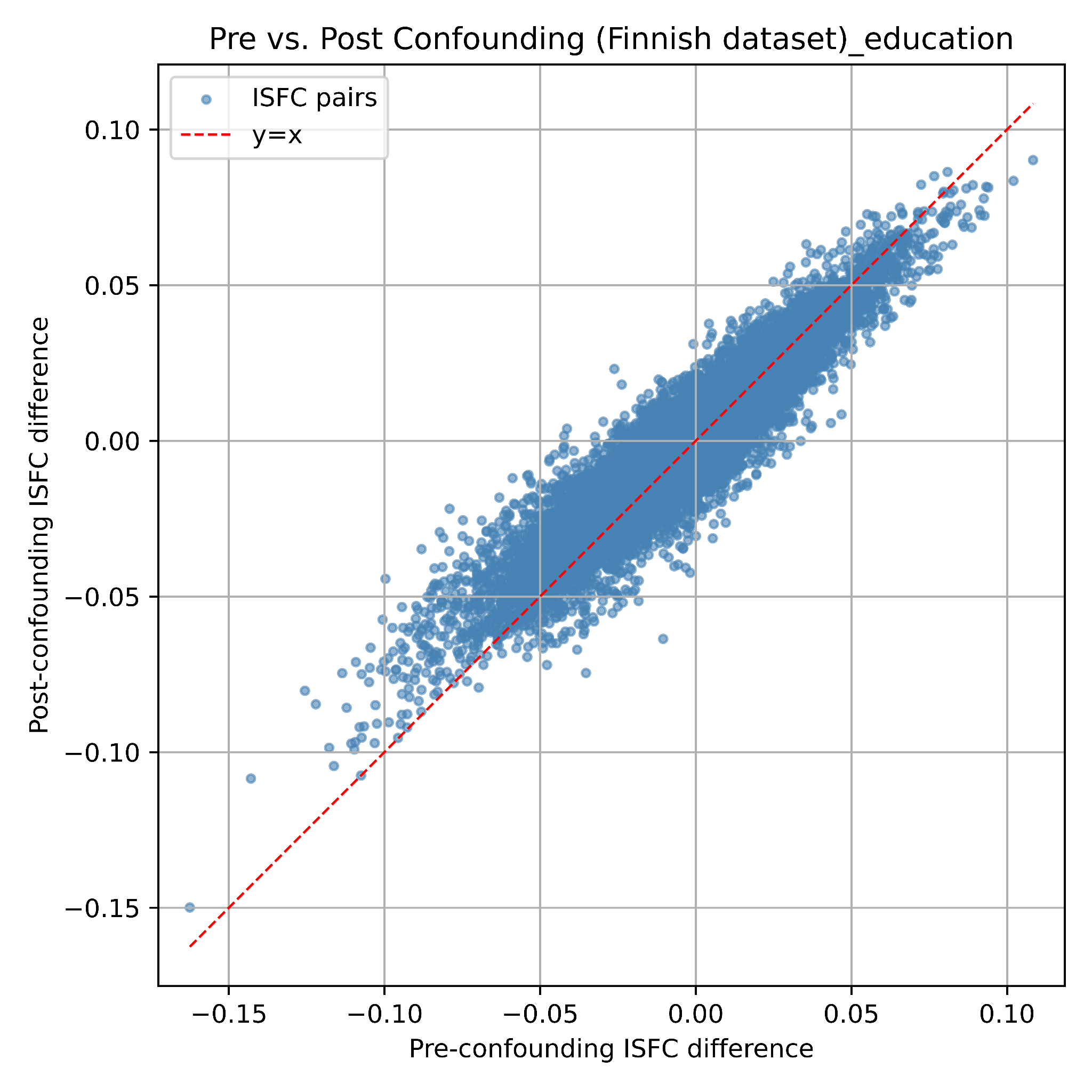
*

**Fig. S4** Scatterplots comparing group differences in ISFC before and after confound regression of education level for the Finnish datasets. Each datapoint represents a median pairwise ISFC group difference (Neurotypical - Autism). The x-axis shows the uncorrected group difference, while the y-axis reflects the group difference after regressing out education levels (pairwise sum and difference). A diagonal line is plotted in red for comparison of pre- and post-regression values.

**References**

(1) Noble KG, Houston SM, Kan E, Sowell ER (2012): Neural correlates of socioeconomic status in the developing human brain. *Developmental science* *15*(4): 516-527.

(2) Noble KG, Engelhardt LE, Brito NH, Mack LJ, Nail EJ, Angal J, et al. (2015): Socioeconomic disparities in neurocognitive development in the first two years of life. *Developmental psychobiology* *57*(5): 535-551.

**Evaluations of head motions on the ISFC and group differences**

To verify that head motion did not introduce spurious stimulus-locked correlations, we evaluated the synchronization of motion time-series across participants. We calculated the pairwise inter-subject correlation (ISC) of Framewise Displacement (FD) for all subject pairs within each subject group (Distributions see Fig. S5A-D). The results showed very low mean correlation (median r < 0.03 in the Finnish dataset and median r < 0.06 in the German dataset) within each sub group (see Fig. S5A-D below). Furthermore, to ensure that motion synchronization did not differ by diagnostic group, we conducted permutation tests (5,000 iterations) comparing the mean motion ISC between the neurotypical and autistic groups in the German and Finnish dataset. As shown in the null distributions (Fig. S5E, F), no significant group differences were observed between the groups (*p* = 0.870 for the German dataset and *p* = 0.544 for the Finnish dataset). These results demonstrate that head motion was unsynchronized across participants and balanced between groups. Given that the ISFC metric mathematically filters out non-synchronized signals (1), these findings strongly suggest that the observed ISFC patterns are driven by neural responses rather than motion-related artifacts in our datasets.

The distributions of group-level motion statistics are visualized below in Fig. S6A. To evaluate the potential group differences of head motion, we did a series of Mann-Whitney U tests across all subject groups. The results indicated no significant difference between the Finnish dataset (mean = 0.14, SD = 0.07) and the German dataset (mean = 0.12, SD = 0.07; *U* = 989.0, *p* = 0.284). Similarly, no significant difference was found between diagnostic categories (*U* = 911.0, *p* = 0.785), with the autistic group (mean = 0.12, SD = 0.06) and the neurotypical group (mean = 0.13, SD = 0.08) showing comparable results. Sub-analyses within each dataset confirmed that autistic and neurotypical groups did not show significantly different motion in either the Finnish (*U* = 177.0, *p* = 0.867) or German (*U* = 289.0, *p* = 0.773) cohorts.

Additionally, to evaluate potential confounding effects of head motions on ISFC group difference, we used linear regression to remove these effects from the ISFC matrices. Specifically, pairwise mean FD and absolute mean FD difference across neurotypical and autistic groups were included as confounders for the German and Finnish dataset. All confound variables were z-scored, and a constant term was added. For each ROI pair, linear models were fit across subject pairs, and confound-related variance was regressed out, resulting in cleaned ISFC matrices that are orthogonal (i.e. independent) to head motions. Next, group-level median ISFC matrices were computed for neurotypical and autistic groups, both before and after confound regression. No notable changes were observed. This was confirmed by a Mantel test comparing pre- and post-regression ISFC group difference matrices, which showed a strong correlation (German dataset: r = 0.98, Finnish dataset: r = 0.99, p < .001, 5000 permutations), indicating that group differences were not primarily driven by individual head motions in two datasets (Fig. S6B, C).

We opted not to use volume censoring since removing timepoints across subject pairs can disrupt the temporal continuity of critical events in the video stimuli and lead to inhomogeneous data loss across the group. Given our current limited sample size, maintaining a consistent timecourse length for all participants was prioritized to avoid spurious correlations and ensure the statistical stability of the ISFC metric (2). Future replication studies with larger sample size and longer movie clips can utilize volume censoring while preserving the temporal sequence if there is significant motion synchronization across participants and groups. In addition, individual head motion should be carefully examined to ensure that ISFC was not reduced to increased inter-subject signal variance.


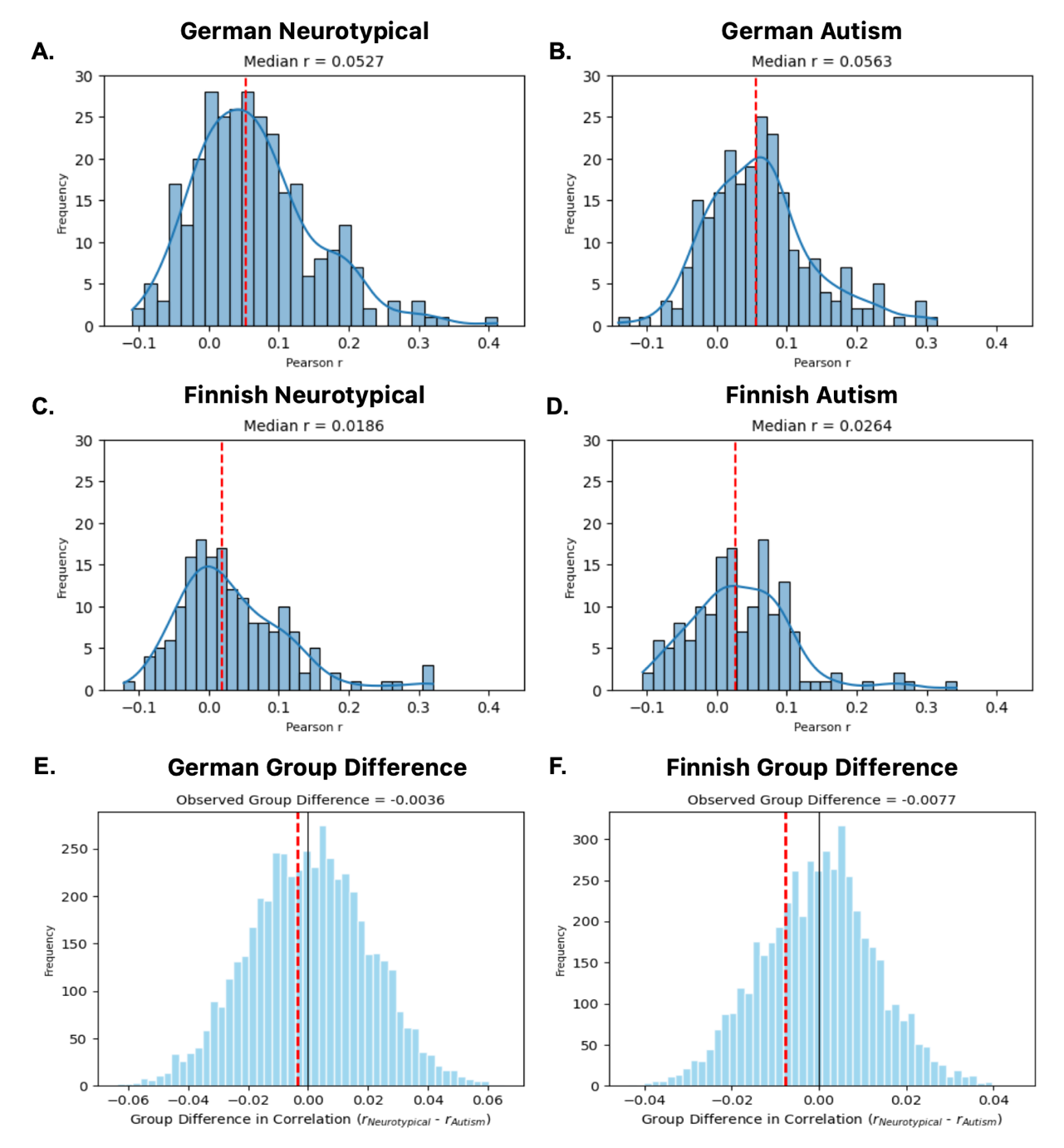


**Fig. S5** Distribution of inter-subject correlation for head motion. Histograms show the frequency of pairwise Pearson correlation coefficients (r) calculated between subjects’ framewise displacement (FD) across all time points for (A) German neurotypical group (B) German autism group (C) Finnish neurotypical group and (D) Finnish autism group. The median r in each group are marked above the frequency plots. The vertical dashed red lines indicate the median correlation of each group. Null distribution of group differences in pairwise motion correlations are visualized in (E) German Neurotypical - Autism and (F) Finnish Neurotypical - Autism. The red dashed lines indicate observed group differences.


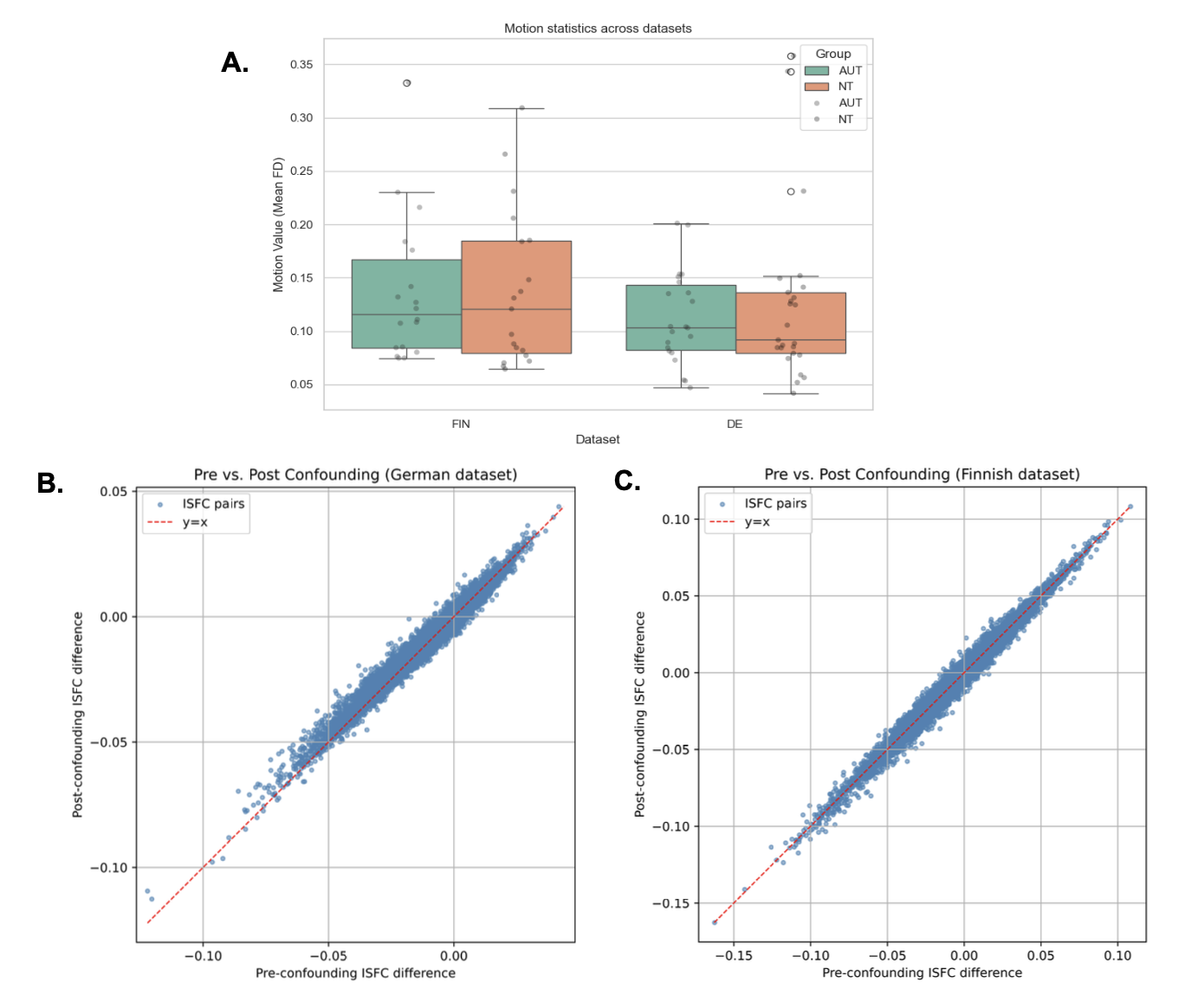


**Fig. S6** Group-level motion statistics and ISFC group differences before and after motion regressions. (A) Box plots of mean FD across four groups (Finnish autistic, Finnish neurotypical, German autistic, German neurotypical). Raw data were plotted as scatters around each box. FIN: Finnish sample; DE: German sample; AUT: Autistic; NT: Neurotypical. Scatterplots comparing group differences in ISFC before and after confound regression of head motions for the (B) German and (C) Finnish datasets. Each datapoint represents a median pairwise ISFC group difference (Neurotypical - Autism) between a pair of brain regions. The x-axis shows the uncorrected ISFC group difference, while the y-axis reflects the group difference after regressing out head motions. A diagonal line is plotted in red for comparison of pre- and post-regression values.

**References**

(1) Simony E, Honey CJ, Chen J, Lositsky O, Yeshurun Y, Wiesel A, et al. (2016): Dynamic reconfiguration of the default mode network during narrative comprehension. *Nature Communications 7*(1):12141.

(2) Lahnakoski JM, Jääskeläinen IP, Sams M, Nummenmaa L (2017): Neural mechanisms for integrating consecutive and interleaved natural events. *Human Brain Mapping 38*: 3360-3376.
